# Biopolymeric superabsorbent hydrogels enhance crop and water productivity of soybean–wheat system in Indo-Gangetic plains of India

**DOI:** 10.1038/s41598-022-16049-x

**Published:** 2022-07-13

**Authors:** G. A. Rajanna, Suman Manna, Anupama Singh, Subhash Babu, V. K. Singh, Anchal Dass, Debashis Chakraborty, Neeraj Patanjali, Indu Chopra, Tirthankar Banerjee, Anil Kumar, Ashish Khandelwal, Balraj S. Parmar

**Affiliations:** 1 ICAR-Directorate of Groundnut Research, RRS, Ananthapur, Andhra Pradesh 515001 India; 2 Division of Agronomy, ICAR-Indian Agricultural Research Institute, New Delhi, 110012 India; 3 ICAR-Central Institute of Fisheries Education, Kolkata Centre, Kolkata, 700091 India; 4 Division of Agricultural Chemicals, ICAR-Indian Agricultural Research Institute, New Delhi, 110012 India; 5 Division of Agricultural Physics, ICAR-Indian Agricultural Research Institute, New Delhi, 110012 India; 6 Division of Design of Experiments, ICAR-Indian Agricultural Statistics Research Institute, New Delhi, 110012 India

**Keywords:** Plant sciences, Environmental sciences

## Abstract

Environmental crises, declining factor productivity, and shrinking natural resource is a threat to global agricultural sustainability. The task is much more daunting in the Indo-Gangetic northern plains of India, where depletion of the underground water table and erratic rains due to the changing climate pose a major challenge to agriculture. To address these challenges a field investigation was carried out during 2016–2018 to test the efficacy of biopolymeric superabsorbent hydrogels namely Pusa Hydrogel (P-hydrogel: a semi-synthetic cellulose derivative-based product) and kaolin derivative of Pusa Hydrogel (K-hydrogel: semi-synthetic cellulose derivative) to assess their effect on crop and water productivity, soil moisture, root dynamics, and economics of soybean (*Glycine max* L.)–wheat (*Triticum aestivum* L.) system under three irrigation regimes namely full irrigation, limited irrigation and rainfed. The results revealed that the full irrigation along with P-hydrogel led to enhanced grain yield, biomass yield, and water productivity (WP) of soybean (1.61–10.5%, 2.2–9.5%, and 2.15–21.8%, respectively) and wheat (11.1–18.3%, 12–54% and 11.1–13.1%, respectively) over control plots. Likewise, under water stressed plots of rainfed conditions with P-hydrogel exhibited 52.7 and 20.6% higher system yields (in terms of wheat equivalent yield) over control and other combinations during the respective study years. Whereas the magnitude of increase in system yield under limited irrigation with P-hydrogel was ~ 15.1% and under full irrigation with P-hydrogel was 8.0–19.4%. Plots treated with P-hydrogel retained 3.0–5.0% higher soil moisture compared to no-hydrogel plots, while K-hydrogel treated plots held the lower moisture (4.0–6.0%) than the control. In terms of profitability, full irrigation along with P-hydrogel plots registered 12.97% higher economic returns over control. The results suggested that P-hydrogel (2.5 kg ha^−1^) reduces runoff water loss in full irrigation applied plots and retained more water, where loss of water is more thus reduces number of irrigations. Hence P-hydrogel with irrigation water is a viable option for sustainable production of soybean-wheat systems in the Indo-Gangetic plains of India and other similar eco-regions of the world.

## Introduction

The growing paucity of water has emerged as the most limiting factor for crop production, particularly in arid- and semi-arid agro-ecologies. In India, the per capita water availability has declined from 5177 m^3^ in 1951 to 1441 m^3^ in 2015 and is expected to decline to 1174 m^3^ by 2051^1^. Serious concerns are being raised over the sustainability of farming techniques involving massive water consumption^2–4^. In such a scenario, precise technologies aiming at reducing consumptive-use (CU) of available water without compromising productivity need to be invented and introduced in crop production. The use of specialty polymers termed superabsorbent or hydrogels have been reported very effective in enhancing retention of the applied water in the soil around the root zone by minimizing percolation and evaporative losses, thus ensuring a better and prolonged supply of moisture to the crop^5,6^. The uses of such materials become more relevant under the conditions of limited water availability such as in arid and semi-arid regions. These materials in granular form hold water and make it available for longer periods through its sustained release to the soil in their zone of application^7–11^.

Hydrogels application improved soil water holding capacity (WHC), resulting in delayed onset of a permanent wilting point under intense evaporation^6,12^. Hydrogels absorb and retain water by 171–402%, mass per mass (m/m)^13^, 80–180 times, m/m^8^, and 67–376 times, m/m^14^ under laboratory conditions. Therefore, the water runoff losses were reduced whereas infiltration rates got enhanced^15^, thus improved soil moisture retention enhanced sorghum biomass yield under rainfed conditions^16^. Improved hydro-physical and chemical conditions of soil through an increase in water-stable soil aggregates and retention pores, decrease in transmission pores and a lowering of soil penetration resistance leads to hydrogel effects^10,17^. Besides the sustained release of water, hydrogels have also been reported to influence nutrient-use efficiency (NUE) by trapping the nutrients in the swollen mass and reducing their losses^9,10^.

The performance of hydrogels depends on the soil and crop types. The addition of polymer in saline soil had positive effects on plant growth, yield, and available moisture content in corn^18^. Likewise, better performance in sandy loam soils over the clay and clay loam soils has been reported^5^. The addition of hydrogels in sandy soil enhanced water availability to plants by reducing drainage loss, increasing retention pores, and reducing soil hydraulic conductivity^19,20^. The light-textured soils characterized by low fertility and moisture deficit resulted in abysmally low crop yields (< 1 to 2 Mg ha^−1^) in drought-prone areas^16^. Crop production in drought-prone areas is constrained largely by variable rainfall conditions. Thus, rainfall variability coupled with drought waves causes 6–14% lower WP in wheat due to higher growth efficiency under the increasing CO_2_ concentration^21^. The WP of cereal crops decreased with climate change due to higher growth period temperature and increased evapotranspiration^22^. Rainfall variability reduces the WP of soybean^22,23^ and wheat^24^.

Various studies reported the beneficial effect of hydrogels in terms of higher soil moisture content and enhanced yields by 12–31% in rice^2,25^, 5–11% in wheat^6^, 31–36% in maize^12^, and 16.4–24.7% in mustard^26–28^. Similarly, water productivity (WP) with hydrogels enhanced by 22.5% in Indian mustard^27^ and 97.1% in maize^29^ under deficit irrigated conditions over no-hydrogel applied plots. Interestingly, hydrogels have also been reported to improve the quality of agricultural produce in terms of fruit and flower size, and color^6^. Despite offering several advantages, the use of hydrogels in agriculture remained very limited mainly because of high application rates (50–225 kg ha^−1^)^30,31^ which incurred higher production cost^17^. Therefore, indigenous biopolymeric polyacrylate hydrogel, P-hydrogel (maximum water-absorbing capacity of 350 times, m/m), and its kaolin based derivative (water-absorbing capacity of 800 times, m/m) was developed for effective moisture conservation at a lower rate of application (2.5–5.0 kg ha^−1^)^32^. Incorporating kaolin with cellulose based anionic polyacrylate as K-hydrogel exhibited higher water-absorbing capacity than P-hydrogel^14,32^.

Depletion of the underground water table is much faster in Indo-Gangetic plains (IGP), mainly due to intense cultivation of high water demanding crops (e.g. rice), changes in cropping pattern (towards more economical crops) coupled with surface water quality reduction^33^. Rice–wheat system is the dominant cropping system in the IGP of India, which requires a lot of water, labor, and energy. Under this situation, shifting towards soybean-wheat cropping systems may be a more economical and water-saving practices in rapidly water declining regions like IGP.

Soybean–wheat cropping, besides being more profitable, is a resource- and energy-use efficient production system^34^. Efficient water use is a major factor in achieving productivity goals in sustainable production systems. Hence, the development of water-saving technology/practices should be a prime focus to the researchers and policy planners designing sustainable agricultural planning. The effect of hydrogels in soybean-wheat systems has not been studied so far. Hence, it was hypothesized that the application of hydrogels may increase soil moisture retention capacity and changes the crop phenology which may improve crop productivity and profitability of soybean-wheat system under various irrigation management practices. Therefore, the present study was conducted with the following objectives, 1) to find out the effect of biopolymeric superabsorbent hydrogels on soil moisture release pattern, rooting behavior, and crop phenology of soybean-wheat system and, 2) to assess the effect of hydrogels on crop and water productivity and profitability of the soybean–wheat system.

## Results

### Soybean seed and biomass yield

A significant effect of irrigation regimes on seed and biomass yields of the soybean was observed during 2017 and 2018 (Table 1). Yields in the full irrigation applied plots (crops do not subjected to drought spells) were significantly higher as compared to rainfed (crops under drought spells) or limited irrigation regimes (crops subjected to short period drought spells). The magnitude of increase being 4.6–9.8% in 2017 and 5.2–12.5% in 2018. The yields differed significantly (P ≤ 0.05) in hydrogel applied treatments also. Maximum seed (1.22–1.37 Mg ha^–1^) and biomass yields (4.9–5.4 Mg ha^–1^) were recorded under P-hydrogel over control and K-hydrogel treatments. Irrigation regimes and hydrogel interaction effects were also significant (Table 1). The marginally higher yield (1.37 and 1.26 Mg ha^–1^) and biomass yields (5.85 and 5.02 Mg ha^–1^) were observed under full irrigation with P-hydrogel during the 1st and 2nd years, respectively. Contrastingly, limited irrigation plots recorded significantly lower seed yields as compared to full irrigation and rainfed plots. However, control plots performed better than K-hydrogel plots with 2–13% and 19–25% higher seed and biomass yields. Among the two tested levels of K-hydrogel, application of K-hydrogel at 2.5 kg ha^−1^ recorded higher seed yield but 5.0 kg ha^−1^ applied plots recorded higher above-ground biomass. Under rainfed regimes also, P-hydrogel and K-hydrogel treatments were superior in terms of seed (15–27%) and biomass yields (36–54%) over control during both the study years.

**Table 1:** Effect of hydrogels and moisture stress conditions on yield of soybean and wheat during 2017–2018 and 2018–2019.

Treatment	Soybean seed yield (Mg ha^−1^)		Soybean biomass yield (Mg ha^−1^)		Wheat grain yield (Mg ha^−1^)		Wheat biomass yield (Mg ha^−1^)		Wheat equivalent yield (Mg ha^−1^)	
	2017	2018	2017	2018	2017–18	2018–19	2017–18	2018–19	2017–18	2018–19
**Irrigation regime (I)**										
Full irrigation	1.16^a^	1.22^a^	5.31^a^	4.81^a^	4.24^a^	5.08^a^	14.90^a^	14.81^a^	6.03^a^	7.33^a^
Limited irrigation	1.11^ab^	1.16^ab^	5.05^ab^	4.79^ab^	3.52^b^	4.54^ab^	12.90^b^	14.20^b^	5.29^b^	6.69^b^
Rainfed	1.05^c^	1.12^c^	4.72^bc^	4.45^c^	2.85^c^	4.09^c^	9.70^c^	13.20^c^	4.56^c^	6.15^c^
**Hydrogel application (H)**										
Control	1.04^c^	1.15^b^	4.87^ab^	4.65^bc^	3.37^b^	4.57^ab^	12.16^a^	14.14^ab^	4.96^bc^	6.70^a^
K-hydrogel @ 2.5 kg ha^–1^	1.11^b^	1.15^bc^	4.76^bc^	4.35^cd^	3.54^b^	4.42^bcd^	12.73^a^	14.09^abc^	5.27^b^	6.54^a^
K-hydrogel @ 5.0 kg ha^–1^	0.98^d^	1.14^bcd^	5.06^ab^	4.87^ab^	3.36^bc^	4.56^abc^	12.22^a^	13.78^cd^	4.86^cd^	6.67^a^
P-hydrogel @ 2.5 kg ha^–1^	1.30^a^	1.22^a^	5.40^a^	4.88^a^	3.88^a^	4.73^a^	12.89^a^	14.28^a^	6.09^a^	6.99^a^
**I × H effect**										
FI + Control	1.24^cd^	1.24^a^	5.82^ab^	5.23^a^	3.78^c^	4.81^c^	13.53^c^	14.04^cd^	5.67^bc^	7.10^b^
FI + K-hydrogel @ 2.5 kg ha^–1^	1.07^f^	1.18^ab^	4.65^cd^	4.38^c^	4.33^ab^	5.07^abc^	15.40^ab^	15.02^ab^	5.92^b^	7.25^ab^
FI + K-hydrogel @ 5.0 kg ha^–1^	0.94^gh^	1.19^ab^	4.91^cd^	4.67^ab^	4.39^ab^	5.11^ab^	15.04^ab^	14.67^b^	5.76^b^	7.31^ab^
FI + P-hydrogel @ 2.5 kg ha^–1^	1.37^a^	1.26^a^	5.85^a^	4.97^ab^	4.47^a^	5.34^a^	15.63^a^	15.53^a^	6.77^a^	7.67^a^
LI + Control	1.07^ef^	1.25^a^	5.41^ab^	5.02^ab^	3.59^cd^	4.59^de^	13.51^c^	14.71^b^	5.24^cd^	6.91^bc^
LI + K-hydrogel @ 2.5 kg ha^–1^	1.29^c^	1.07^c^	5.03^cd^	4.63^ab^	3.32^d^	4.19^ef^	12.73^cd^	14.24^c^	5.36^c^	6.16^ef^
LI + K-hydrogel @ 5.0 kg ha^–1^	0.84^hi^	1.14^b^	4.65^cd^	4.86^ab^	3.24^d^	4.75^cd^	12.45^de^	13.86^cde^	4.55^e^	6.84^cd^
LI + P-hydrogel @ 2.5 kg ha^–1^	1.22^b^	1.19^ab^	5.11^cd^	4.67^ab^	3.94^bc^	4.63^d^	12.93^c^	14.00^cd^	6.03^b^	6.83^cd^
RF + Control	0.79^j^	0.95^d^	3.39^de^	3.69^cde^	2.73^f^	4.31^e^	9.44^fg^	13.67^ef^	3.95^fg^	6.08^ef^
RF + K-hydrogel @2.5 kg ha^–1^	0.98^fg^	1.19^ab^	4.61^cd^	4.05^cd^	2.99^ef^	4.00^fg^	10.06^f^	13.01^fg^	4.53^e^	6.20^ef^
RF + K-hydrogel @5.0 kg ha^–1^	1.14^de^	1.10^b^	5.62^ab^	5.07^ab^	2.44^fg^	3.81^gh^	9.19^f^	12.82^gh^	4.27^ef^	5.85^fg^
RF + P-hydrogel @2.5 kg ha^–1^	1.31^ab^	1.22^a^	5.24^b^	5.01^ab^	4.24^a^	5.08^a^	14.90^a^	14.81^a^	6.03^a^	7.33^a^

Means followed by a similar superscript letter within a column are not significantly different (at P ≤ 0.05) between treatments allowing to least significant difference test. Superscripts denotes the superior of the treatments i.e.^a^denotes best treatment followed by next best treatment^b^ and so on.

*FI* full irrigation, *LI* limited irrigation, *RF* rainfed; gel, hydrogel.

### Wheat grain and biomass yield

The irrigation regimes had a significant effect on wheat grain and biomass yields (Table 1). The highest grain (4.24–5.08 Mg ha^–1^) and biomass yields (14.90–14.81 Mg ha^–1^) were obtained under full irrigation plots as compared to the limited irrigation (grain yield, 3.52–4.54 Mg ha^–1^; biomass yield, 12.90–14.20 Mg ha^–1^) and rainfed regime (grain yield, 2.85–4.09 Mg ha^–1^; biomass yield, 9.70–13.20 Mg ha^–1^). The grain and biomass yields increased significantly (P ≤ 0.05) with P-hydrogel application, the magnitude of increase being 3.0–15.0 and 2.0–6.0% over control, respectively. However, control plots exhibited slightly higher (~ 2%) seed and biomass yields over K-hydrogel (both at 5.0 and 2.5 kg ha^–1^) but differences were non-significant. The interaction effect of hydrogels and irrigation regimes on wheat yields was significant in both the study years. Full and limited irrigations with P-hydrogel treatments led to an increase in grain yield (11–18%) and biomass yield (1.2–9.8%) as compared to control. Limited irrigations and rainfed plots with no–hydrogel registered significantly higher grain (9–11% and 3–7%) and biomass yields (4–9% and 2–6%) as compared to K-hydrogel treatments, respectively.

### Soybean-wheat system yields

Irrigation regimes expressed a significant influence on wheat equivalent yield (Table 1). During both, study years, the wheat equivalent yield (WEY) was significantly higher under full irrigation (10–32%) as compared to the limited irrigation and rainfed regimes. The effect of hydrogel application on WEY was significant in 2017–18, where the application of P-hydrogel caused a significant increase in WEY (~ 22%) over control. In 2018–19, the treatment differences were non-significant. A positive and significant (P ≤ 0.05) interaction effect of irrigation regimes and hydrogels on WEY was observed during both the study years. The WEY in P-hydrogel with full irrigation (5.67–6.77 Mg ha^–1^ in 2017–18 and 7.10–7.67 Mg ha^–1^ in 2018–19) was significantly higher as compared to the other combinations. However, the WEY was 8–19% lower under K-hydrogel (5.0 kg ha^–1^) applied plots over control.

Likewise, under water stressed plots of rainfed conditions with P-hydrogel exhibited 52.7 and 20.6% higher system yields (in terms of wheat equivalent yield) over control and other combinations during the respective study years. Whereas the magnitude of increase in system yield under limited irrigation with P-hydrogel was ~ 15.1% and under full irrigation with P-hydrogel was 8.0–19.4%.

### Water productivity (WP) and irrigation water productivity (IWP)

In soybean, WP (8.1–21.0%) and IWP (126–817%) were significantly higher in rainfed regimes compared to those in full and limited irrigations, respectively (Table 2). Among hydrogels, the application of P-hydrogel resulted in a significant increase in WP (7–41%) and IWP (2–22%) over control. Interaction effects of irrigation regimes and hydrogels for the two years found significant, full, and limited irrigations with P-hydrogel exhibited the highest WP (3–22%) and IWP (1.2 to 5.0%) over control. However, lower WP (9–15%) and IWP (5–17%) were recorded under K-hydrogel applied plots over control plots.

**Table 2:** Effect of hydrogels and moisture stress conditions on water productivity (WP) and irrigation water productivity (IWP) in soybean–wheat during 2017–19.

Treatment	Soybean				Wheat			
	WP (kg ha-cm^−1^)		IWP (kg ha-cm^−1^)		WP (kg ha-cm^–1^)		IWP (kg ha-cm^−1^)	
	2017	2018	2017	2018	2018	2019	2018	2019
**Irrigation regime (I)**								
Full irrigation	21.3^c^	18.3^bc^	67.8^c^	121.7^c^	150.2^c^	176.6^c^	169.7^c^	254.1^c^
Limited irrigation	23.6^b^	18.9^b^	100.9^b^	232.7^b^	193.0^b^	241.8^b^	234.7^b^	454.0^b^
Rainfed	25.8^a^	19.8^a^	194.3^a^	1116.5^a^	345.9^a^	312.1^a^	570.0^a^	860.0^a^
Hydrogel application (H)								
Control	21.0^c^	18.7^b^	101.6^d^	443.3^d^	220.6^bc^	241.4^b^	312.1^bc^	520.5^b^
K-hydrogel @ 2.5 kg ha^–1^	23.1^b^	18.8^ab^	117.2^b^	508.1^ab^	232.5^ab^	229.9^cd^	330.5^b^	490.8^bc^
K-hydrogel @ 5.0 kg ha^–1^	20.4^d^	18.6^bc^	111.5^c^	482.4^bc^	209.8^cd^	235.7^bc^	293.4^cd^	497.5^cd^
P-hydrogel @ 2.5 kg ha^–1^	29.7^a^	20.0^a^	153.6^a^	527.5^a^	255.8^a^	266.9^a^	363.2^a^	582.0^a^
**I × H effect**								
FI + Control	22.5^d^	18.6^de^	71.6^ghi^	123.9^f^	134.0^ij^	167.1^fg^	151.3^gh^	240.5^fg^
FI + K-hydrogel @ 2.5 kg ha^–1^	19.1^e^	17.8^ef^	60.6^hij^	118.0^fg^	153.2^ghi^	176.2^f^	173.0^g^	253.5^f^
FI + K-hydrogel @ 5.0 kg ha^–1^	16.3^ef^	17.9^ef^	51.8^j^	119.0^f^	155.4^ghi^	177.6^f^	175.6^g^	255.5^f^
FI + P-hydrogel @ 2.5 kg ha^–1^	27.4^b^	19.0^d^	87.1^fg^	125.9^f^	158.2^ghi^	185.6^f^	178.8^f^	267.0^f^
LI + Control	22.1^d^	20.5^abc^	94.2^f^	251.4^e^	196.6^ef^	244.4^d^	239.0^ef^	459.0^de^
LI + K-hydrogel @ 2.5 kg ha^–1^	27.1^bc^	17.4^ef^	115.7^e^	214.0^e^	182.0^efg^	223.1^e^	221.4^ef^	418.5^e^
LI + K-hydrogel @ 5.0 kg ha^–1^	17.4^e^	18.5^de^	74.4^gh^	227.0^e^	177.5^efgh^	252.9^d^	215.8^ef^	474.8^d^
LI + P-hydrogel @ 2.5 kg ha^–1^	27.9^b^	19.4^cd^	119.0^e^	238.4^e^	216.0^e^	246.5^d^	262.6^e^	463.0^de^
RF + Control	18.4^e^	16.9^fg^	138.9^d^	954.7^d^	331.4^bc^	312.8^a^	546.1^bc^	862.9^a^
RF + K-hydrogel @2.5 kg ha^–1^	23.2^d^	21.1^ab^	175.3^c^	1192.3^ab^	362.3^ab^	290.3^bc^	597.1^ab^	800.0^bc^
RF + K-hydrogel @5.0 kg ha^–1^	27.6^b^	19.5^cd^	208.3^b^	1101.1^c^	296.5^cd^	276.5^c^	488.7^cd^	762.2^c^
RF + P-hydrogel @2.5 kg ha^–1^	33.8^a^	21.6^a^	254.6^a^	1218.1^a^	393.3^a^	368.7^ab^	648.2^a^	1016.1^b^

Means followed by a similar superscript letter within a column are not significantly different (at P ≤ 0.05) between treatments allowing to least significant difference test. Superscripts denotes the superior of the treatments i.e.^a^denotes best treatment followed by next best treatment^b^ and so on.

*FI* full irrigation, *LI* limited irrigation, *RF* rainfed.

The WP and IWP of wheat were higher by 41–213% and 49–311% in rainfed plots as compared to the full and limited irrigations, respectively during 2017–18 and 2018–19. Unlike irrigation regimes, the hydrogel application did not affect the WP and IWP of wheat significantly during 2017–2018 (Table 2), however in 2018–19, WP and IWP were maximum in P-hydrogel applied plots (252 and 457 kg ha-cm^–1^, respectively) which were significantly higher by 4.9% over the other K-hydrogel and control plots. Interestingly, K-hydrogel applied at both levels (5.0 and 2.5 kg ha^–1^) recorded 5–12 and 6–14% lower WP and IWP as compared to control plots, respectively.

### Wheat phenological effects

**Figure 1:**
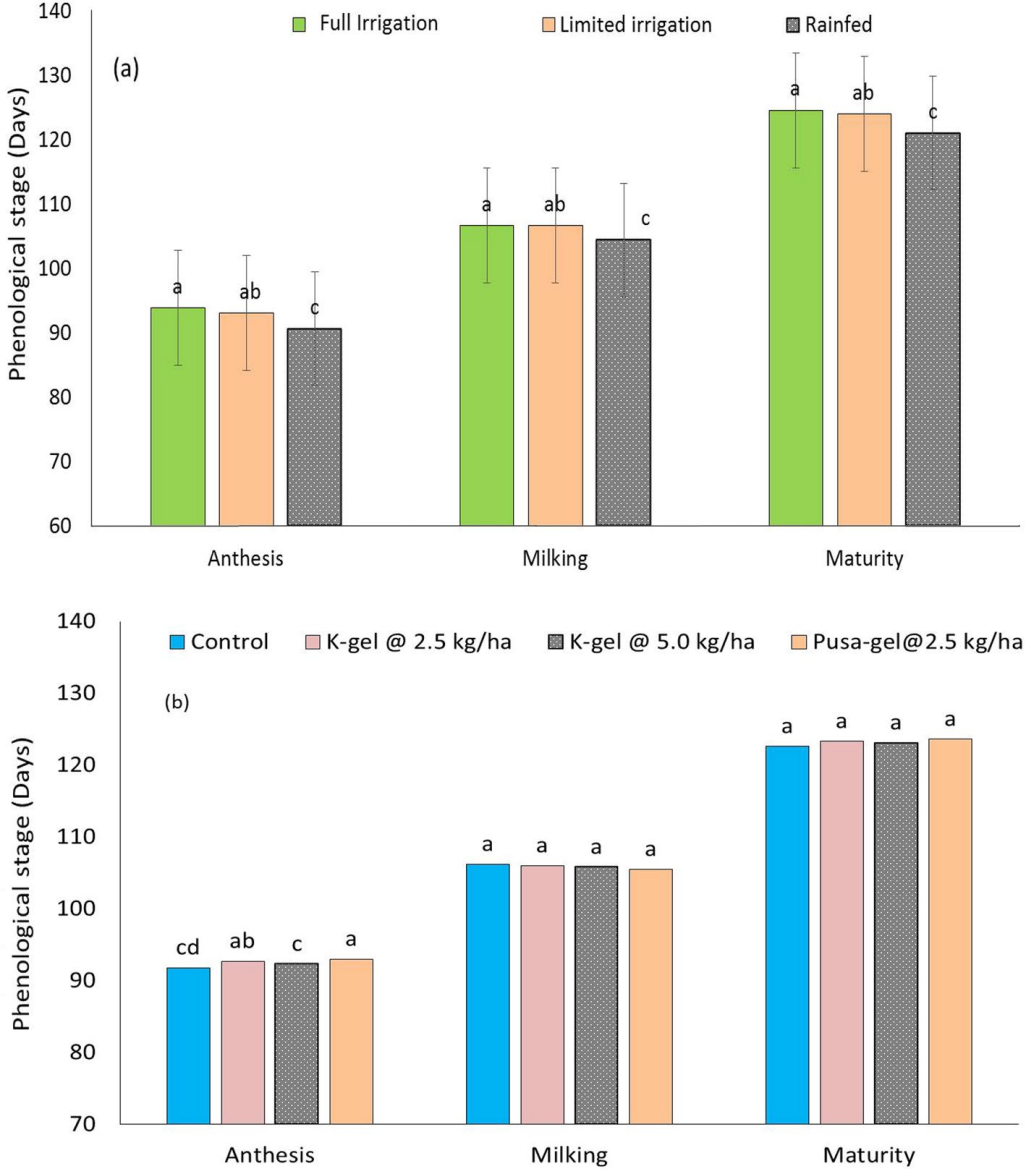
Effect of irrigation regimes (**a**) and hydrogels (**b**) on phenology of wheat. Bars indicate standard error of mean.

Significant (P ≤ 0.05) effects of irrigation regimes and hydrogels on wheat phenology were observed during 2018–19 (Fig. 1). Wheat crop took 2–3 days more to attain anthesis (93.8 days), milking (106.6 days), and maturity (124.5 days) over the rainfed plots (90.6, 104.4, and 121.0 days, respectively). Among hydrogels, wheat took a significantly greater number of days to attain anthesis (93.0 days) in P-hydrogel applied plots over control (91.8 days), K-hydrogel @ 2.5 (92.7 days), and K-hydrogel @ 5.0 kg ha^−1^ (92.3 days). A non-significant effect of hydrogels was, however, observed for days to milking and maturity.

### Wheat root length and volume

**Figure 2:**
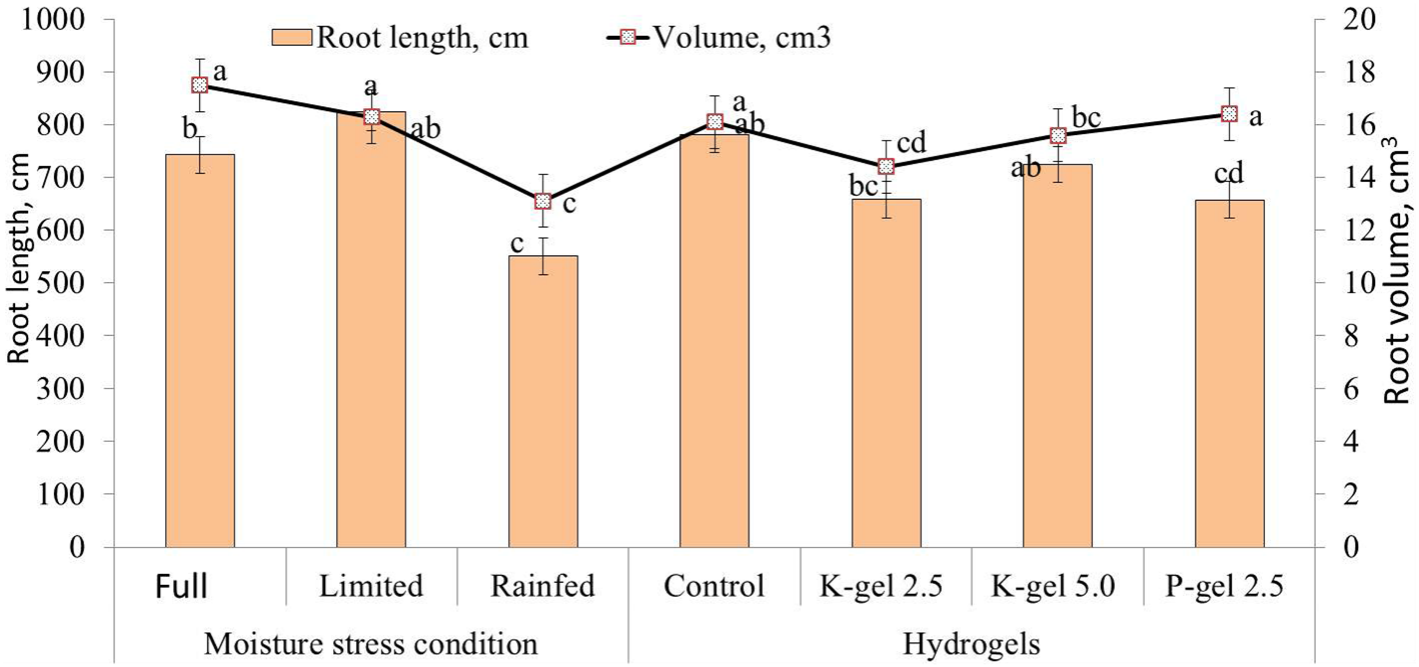
Individual effect of hydrogels and irrigation regimes on root length and volume of wheat in 2018–19. Bars indicate standard error of mean.

**Figure 3:**
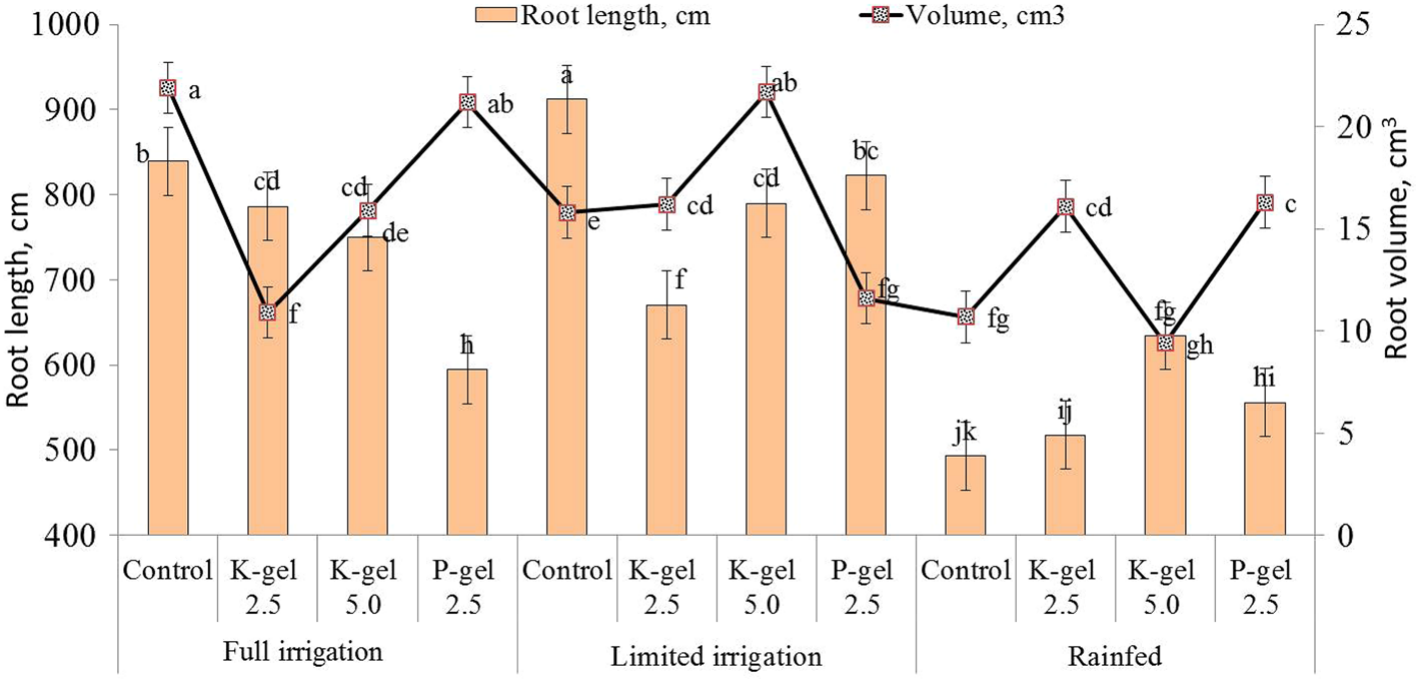
Combined effects of hydrogels and irrigation regimes on root length and volume of wheat in 2018–19. Bars indicate standard error of mean.

The total root length and volume of wheat were recorded for different irrigation regimes during 2018–19 (Fig. 2). Appreciably higher root length and volume under full (742.7 cm and 17.5 cm^3^, respectively) and limited irrigation plots (823.8 cm and 16.3 cm^3^, respectively), were observed as compared to rainfed plots (550.4 cm and 13.1 cm^3^, respectively). Hydrogels also showed a significant (P ≤ 0.05) effect on root length and volume of wheat (Fig. 2). Control plots exhibited significantly more root length (781.6 cm) and volume (16.1 cm^3^) of wheat as compared to K-hydrogel and P-hydrogel. However, P-hydrogel plots showed significantly lower root length (657.7 cm) and higher root volume (16.4 cm^3^) as compared to others. Among the interaction effects, control plots under both full and limited irrigations exhibited significantly higher root length and volume as compared to hydrogel applied plots (Fig. 3). While in rainfed regimes, K-hydrogel @ 5.0 kg ha^−1^ recorded the highest root length and volume over others.

### Soil moisture dynamics in wheat

**Figure 4:**
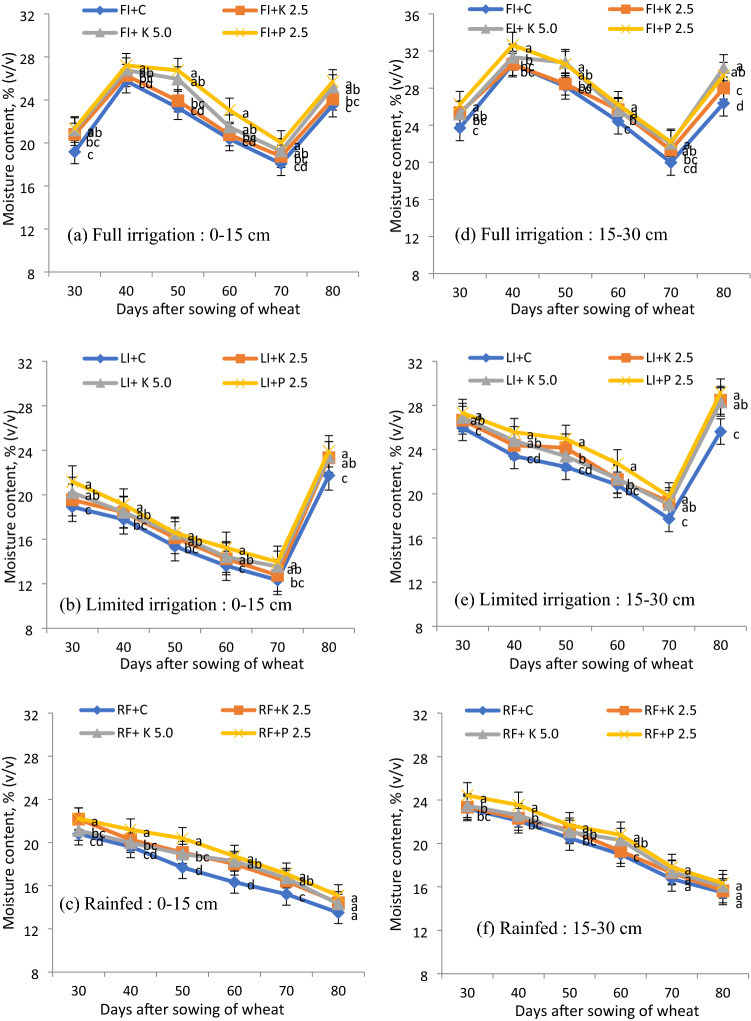
Volumetric water content (v/v) in wheat under different hydrogels and irrigation regimes at 0–15 (**a–c**) and 15–30 (**d–f**) cm soil depths during 2017–2018. Bars indicate standard error of mean. *FI* full irrigation, *LI* limited irrigation, *RF* rainfed; gel, hydrogel. Means followed by a similar superscript letter within a column are not significantly different (at P ≤ 0.05) between treatments allowing to least significant difference test.

**Figure 5:**
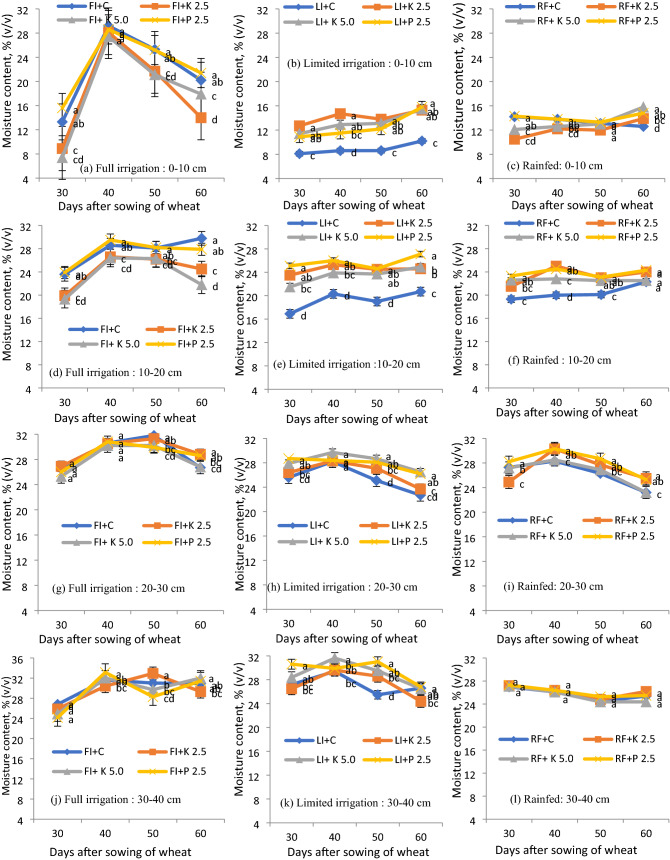
Volumetric water content (v/v) in wheat under different hydrogels and moistures stress conditions at 0–10 (**a–c**), 10–20 (**d–f**), 20–30 (**g–i**) and 30–40 (**j–l**) cm soil depths during 2018–19. Bars indicate standard error of mean. *FI* full irrigation, *LI* limited irrigation, *RF* rainfed, *gel* hydrogel. Means followed by a similar superscript letter within a column are not significantly different (at P ≤ 0.05) between treatments allowing to least significant difference test.

The volumetric soil moisture (v/v) variation at 0–15 cm and 15–30 cm depth was observed in 2017–18 (Fig. 4) and at 0–10 cm, 10–20 cm, 20–30 cm, and 30–40 cm depth in 2018–19 (Fig. 5). During 2017–18, at all irrigation regimes, applying hydrogels caused a slight increase in soil moisture for 0–15 cm and 15–30 cm soil profile depths (Fig. 4). Further, P-hydrogel applied plots had significantly higher soil moisture content by 3–5% than control plots. K-hydrogel @ 2.5 and 5.0 kg ha^–1^ applied plots showed almost equal soil moisture content at all the growth stages of wheat but it was slightly lower than control plots. During 2018–2019, the soil moisture content showed higher values for P-hydrogel applied plots for all depths (0–10 cm, 10–20 cm, 20–30 cm, and 30–40 cm) (Fig. 5), with a significant increase at the level of 0–20 cm at all the growth stages of wheat where P-hydrogel was incorporated. But the soil moisture content at lower-profile depths of 20–30 and 30–40 cm was non-significant. In terms of pressure plate apparatus, in the first year, the soil moisture content in P-hydrogel plots was higher in certain observation days, e.g., 30, 40, and 60 DAS (limited) and nearly all the DAS (rainfed) in 0–15 cm layer; 40, 50 and 60 DAS (limited) and 40 and 60 DAS (rainfed) in 15–30 cm layers.

### Water retention-release pattern in hydrogels

**Figure 6:**
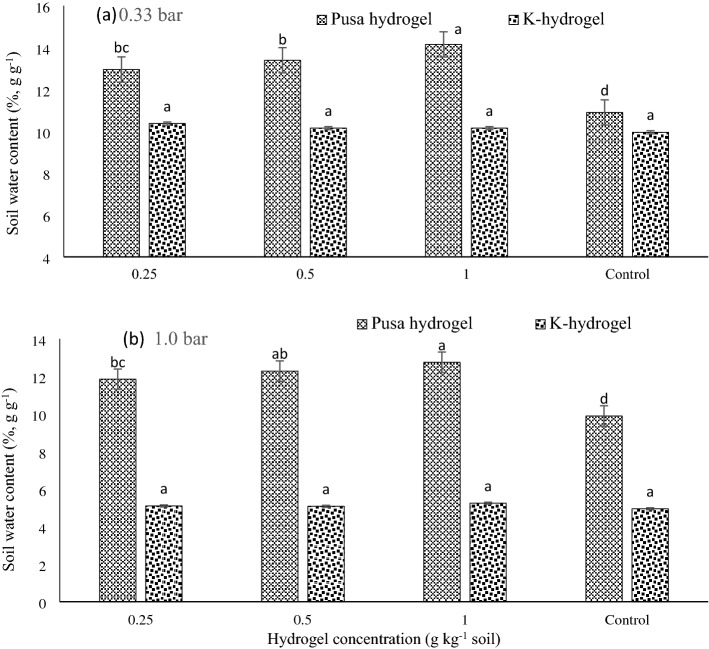
Pressure plate apparatus determined soil moisture retention release [(**a**) 0.33 bar and (**b**) 1.0 bar] under P- and K-hydrogels under different hydrogel concentreations. Bars indicate standard error of mean.

Moisture retention by the two hydrogels, each at two application rates, was studied at 0.33 and 1.0 bars. At both the pressure points, P-hydrogel retained a significantly higher amount of water compared to K-hydrogel and control (no-hydrogel). P-hydrogel held more water at suction 0.33 bar (field capacity) as compared to control and K-hydrogel. K-hydrogel releases more water (~ 5%, v/v) compared to P-hydrogel (2–3%, v/v), and also the P- hydrogel has higher retention at 1 bar indicating its effectiveness is limited in dry soil water condition. While P-hydrogel still has the potential to hold significantly more water and exhibit greater residual soil moisture (available for plants) over K-hydrogel and control (Fig. 6). Whereas, K-hydrogel had a non-significant effect on soil moisture retention and release pattern as compared to control.

### Profitability analysis of soybean–wheat system

ANOVA revealed a significant effect of irrigation regimes and hydrogels on soybean and wheat profitability during 2018–19 (Table 3). The Gross and net profitability of soybean was significantly (P ≤ 0.05) higher by 4.1–5.9% and 5.8–12.0% in full irrigation applied plots over the rainfed regime and limited irrigation, respectively. In wheat, there was a 20–49% enhancement in gross profitability and 37–109% in net profitability due to full irrigation over limited irrigation and rainfed regimes. Also, significant increases in wheat gross profitability (5–15%) and net profitability (8–18%) were found with the application of P-hydrogel compared to no- hydrogel and K-hydrogel. While in both soybean and wheat, K-hydrogel at 5.0 kg ha^–1^ resulted in significantly lower net profitability by 3–16% over control. Benefit: cost ratio (B:C) in soybean did not differ significantly with irrigation regimes, however, the effect of hydrogel application was significant. A higher B:C ratio of 2.38 was recorded under full irrigations than the other irrigation regimes. Among hydrogels, a significantly higher B:C ratio was recorded in control plots (1.72 and 2.38) for soybean and wheat over K-hydrogel but it was at-par with P-hydrogel plots.

**Table 3:** Effect of hydrogels and moisture stress conditions on economics of soybean and wheat production in 2018–19.

Treatment	Soybean				Wheat			
	Cost of production (US$ ha^−1^)	Gross profitability (US$ ha^−1^)	Net profitability (US$ ha^−1^)	B:C	Cost of production (US$ ha^−1^)	Gross returns (US$ ha^−1^)	Net returns (US$ ha^−1^)	B:C
**Irrigation regime (I)**								
Full irrigation	377.3^a^	608.1^a^	230.7^a^	1.62^a^	570.6^a^	1359.4^a^	788.8^a^	2.38^a^
Limited irrigation	364.3^a^	582.6^b^	218.3^ab^	1.61^a^	544.4^a^	1216.3^b^	671.9^b^	2.24^b^
Rainfed	351.2^a^	558.0^c^	206.8^bc^	1.59^a^	518.2^a^	1096.3^c^	578.1^c^	2.12^c^
**Hydrogel application (H)**								
Control	333.9^a^	575.3^b^	241.4^ab^	1.72^a^	514.0^a^	1224.7^ab^	710.7^ab^	2.38^a^
K-hydrogel @ 2.5 kg ha^–1^	363.8^a^	571.9^bc^	208.1^c^	1.57^bc^	544.0^a^	1185.1^bc^	641.1^bc^	2.17^b^
K-hydrogel @ 5.0 kg ha^–1^	393.7^a^	573.0^b^	179.3^cd^	1.46^d^	573.9^a^	1219.7^b^	645.8^b^	2.12^bc^
P-hydrogel @ 2.5 kg ha^–1^	365.6^a^	611.4^a^	245.8^a^	1.67^ab^	545.7^a^	1266.5^a^	720.8^a^	2.32^ab^
**I × H effect**								
FI + Control	347.0^a^	621.3^ab^	274.3^ab^	1.79^ab^	540.2^a^	1285.8^bc^	745.6^bc^	2.38^ab^
FI + K-hydrogel @ 2.5 kg ha^–1^	376.9^a^	587.5^bcd^	210.6^cde^	1.56^def^	570.1^a^	1357.3^ab^	787.1^ab^	2.38^ab^
FI + K-hydrogel @ 5.0 kg ha^–1^	406.8^a^	594.4^ab^	187.5^de^	1.46^ef^	600.1^a^	1365.9^ab^	765.8^ab^	2.28^abc^
FI + P-hydrogel @ 2.5 kg ha^–1^	378.7^a^	629.1^a^	250.4^bc^	1.66^cd^	571.9^a^	1428.7^a^	856.8^a^	2.50^a^
LI + Control	333.9^a^	628.2^ab^	294.3^a^	1.88^a^	514.0^a^	1231.6^cde^	717.6^bc^	2.40^ab^
LI + K-hydrogel @ 2.5 kg ha^–1^	363.8^a^	537.3^ef^	173.5^ef^	1.48^ef^	544.0^a^	1124.7^f^	580.7^e^	2.07^d^
LI + K-hydrogel @ 5.0 kg ha^–1^	393.7^a^	569.6^d^	175.8^ef^	1.45^f^	573.9^a^	1269.5^c^	695.6^bc^	2.21^bcd^
LI + P-hydrogel @ 2.5 kg ha^–1^	365.6^a^	595.2^ab^	229.6^cd^	1.63^cde^	545.7^a^	1239.2^cd^	693.5^cd^	2.27^bcd^
RF + Control	320.8^a^	476.3^g^	155.5^fg^	1.48^fg^	487.9^a^	1156.7^def^	668.8^cd^	2.37^ab^
RF + K-hydrogel @2.5 kg ha^–1^	350.7^a^	590.9^bc^	240.1^bc^	1.68^bc^	517.8^a^	1073.3^f^	555.5^ef^	2.07^d^
RF + K-hydrogel @5.0 kg ha^–1^	380.7^a^	555.1^de^	174.4^ef^	1.46^ef^	547.7^a^	1023.8^g^	476.0^fg^	1.87^e^
RF + P-hydrogel @2.5 kg ha^–1^	352.5^a^	609.8^abc^	257.3^bc^	1.73^bc^	519.6^a^	1131.7^f^	612.1^de^	2.18^cd^

Means followed by a similar superscript letter within a column are not significantly different (at P ≤ 0.05) between treatments allowing to least significant difference test. Superscripts denotes the superior of the treatments i.e.^a^denotes best treatment followed by next best treatment^b^ and so on.

*FI* full irrigation, *LI* limited irrigation, *RF* rainfed, *B:C* benefit: cost.

## Discussion

### Irrigation effects on productivity, phenology, root attributes and soil moisture dynamics

More stable and significantly higher soybean and wheat yields were recorded under adequate irrigation (crops do not subjected to drought spells) during both the study years (2017–18 and 2018–19); grain and biomass yields of soybean and wheat were increased significantly by 4.6–9.8% and 5.2–12.5%, and 24–49% and 12–54%, respectively under adequate irrigation compared to rainfed plot (crops under frequent drought spells) due to better growth and development of soybean and wheat crops observed owing to favorable soil moisture content under full irrigation regimes. In frequent and adequate irrigation applied plots the surface layers remained wet for a longer duration, maintaining favorable conditions during flowering to maturity time resulting in higher water and nutrient uptake^28,35,36^ and finally enhanced yield parameters and yield^37,38^ compared to limited-irrigated (crops subjected to short period drought spells) or rainfed plots (crops under frequent drought spells). Such enhancements in soybean and wheat yields caused a 10–32% improvement in system productivity. Concurrently, wheat crop took 2–3 days more to attain anthesis, milking, and maturity under full and limited irrigations over rainfed plots due to optimum moisture regime favoring continued photosynthesis, plant growth, and delaying its life cycle^35,36^.

**Figure 7:**
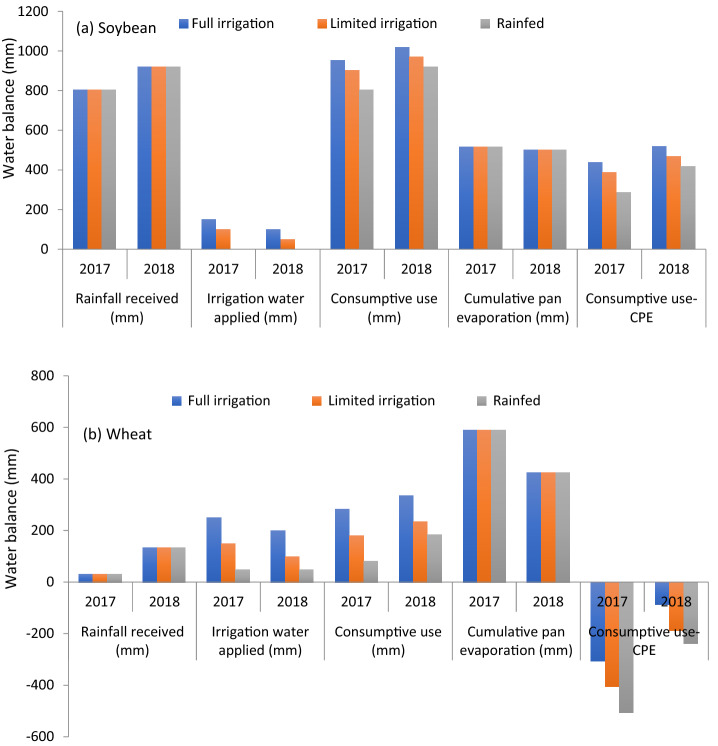
Water balance sheets of (**a**) soybean and (**b**) wheat during 2017–2018 and 2018–2019.

The rate of carbon assimilation over transpired water is denoted as WUE, which acts as a bridge between the carbon and water cycle in agro-ecosystems^39^. In the current investigation, a higher WP of soybean and wheat was observed under rainfed conditions compared to ample or even limited irrigation, involving a much higher amount of water-use without showing proportionate yield increments. Parihar et al.^40^ have also earlier reported that soil moisture conserved in the seed-zone not only provided better crop establishment and growth but also increased WP. Incidentally, relatively more frequent irrigations scheduled at 40% depletion of available soil moisture in full irrigated plots resulted in higher soil moisture content at all the crop growth stages. Positive impact on water balance (consumptive use-cumulative pan evaporation, CPE) of soybean was much higher during 2018 over 2017 due to higher rainfall and lower irrigation water application. However, the impact on water balance (consumptive use-cumulative pan evaporation, CPE) of wheat was much higher during 2017 over 2018 (Fig. 7).

Appreciably higher root length and volume under full and limited irrigation plots were observed as compared to rainfed plots (Figs. 2 and 3). In irrigated plots, the roots were concentrated in the upper layer and had greater horizontal development, which might be due to better moisture availability^28,36^. In rainfed plots, root length and volume were negatively affected due to relatively deficient moisture conditions, where roots did not proliferate as much as under full irrigation and hence were not able to extract water from deeper layers. Higher root mass and density in deeper soil layers enhance the water extraction capacity for increased wheat yield under terminal drought stress^41^. While, large root mass may aggravate water stress in the topsoil layer thus reducing stomatal conductance and photosynthesis^42^.

### Hydrogel effects on productivity, phenology, root attributes and soil moisture dynamics

Hydrogels have been used as water-retaining polymers in agriculture^2,6,16,25–27^ since they can retain a great amount of water when incorporated in soil and release it slowly more or less matching with plant requirement leading to improved crop growth and yield under water-stressed conditions^43^. In the current study, the applied hydrogels exhibited a significant effect on soybean and wheat growth, especially under limited irrigation and rainfed conditions. A significant (P ≤ 0.05) enhancement in soybean seed yield (6–25%) and wheat grain yield (3–15%) in P-hydrogel applied plots compared to no–hydrogel (control) and K-hydrogel applied plots were observed (Table 1). All the other parameters being constant, the increase in yield may be attributed to the extended availability of water to plants in the polymer treatments during periods of water stress^10,11,43^. Similarly, Jarvis and Davies^44^ reported that the increased photosynthesis rate and leaf relative water content in plants under superabsorbent polymers would enhance growth under drought-stress conditions. Our results are consistent with earlier studies which showed that higher crop yields were attained under hydrogel application in rice^2,25^, wheat^6^, maize^12^ and mustard^26,27^.

Islam et al.^29^and Shekari et al.^37^ studied water and yield interaction and concluded that the polymers enhanced the WHC of the soils, which is more beneficial for enhancing water and nutrient uptake by wheat and thus leads to higher above-ground biomass. They found that there was a significant effect of irrigation regimes and superabsorbent polymers on total above-ground biomass and water productivity. Our results also suggested that the application of P-hydrogel @ 2.5 kg ha^–1^ resulted in a significant increase in WP and IWP over no–hydrogel plots (Table 2). Thus, the application of P-hydrogel to the soil surface helped in retaining higher soil moisture in the root zone, and successive slow release of moisture led to crop yield enhancement with lesser water consumption. In contrast, K-hydrogel at 5.0 and 2.5 kg ha^–1^ recorded 5–12 and 6–14% lower WP and IWP compared to no-hydrogel plots. This could be due to proportionately more loss of water in evapotranspiration, or slow or no release of water in K-hydrogel applied plots. The difference in the relative retention release ratios observed under rainfed and full irrigated conditions can be understood in terms of macromolecular expansion in presence of plenty of water (full irrigation and high rains), which led to higher water absorption and retention. Contrastingly, K-hydrogel releases more water (~ 5%, v/v) compared to P- hydrogel (2–3%, v/v), thus the released water was prone to evaporation losses. The presence of limited water with less rainfall or limited irrigated conditions led to suboptimal release absorption.

Phenology is an important criterion to decide the yield potential of any crop^36^. Underwater stress conditions any reduction in the number of days taken to attain different phenophases of the wheat will greatly affect the yield potential. The same was reflected in this study, where wheat took a significantly greater number of days to attain anthesis (93.0 days) in P-hydrogel applied plots over no-hydrogel and K-hydrogel (Fig. 1). Thus, P-hydrogel favored wheat plant growth owing to better soil moisture regimes and enhanced root-attributes. A non-significant effect of hydrogels was, however, observed for days to milking and maturity.

Hydrogels also showed a significant effect on root length and volume of wheat during 2019 (Fig. 2). No-hydrogel plots exhibited significantly (P ≤ 0.05) higher root length (781.6 cm) and volume (16.1 cm^3^) of wheat as compared to K-hydrogel and P-hydrogels. Due to water stress, roots explored lower horizons and had a vertical distribution in the lower moisture regimes and in control plots. However, P-hydrogel applied plots showed significantly lower root length and higher root volume (Fig. 3) because of better soil moisture availability to wheat plants, thus enhancing their water stress tolerance capacity and finally leading to better crop yields. However, Rezashateri et al.^45^ reported that hydrogels seemed to increase root growth and decrease irrigation frequency initially for crop plants.

It is evident from Figs. 4 and 5 that application of hydrogels improved the soil water content under all the irrigation regimes, and eventually, there was an improvement in growth, yield, and higher WP (Tables 1 and 2). Further, P-hydrogel applied plots had 3–5% higher soil moisture content than no-hydrogel applied plots. Hydrogel applied plots had similar moisture release patterns with no-hydrogel plots, but the amount of WHC varied among the hydrogels and it was slightly higher under P-hydrogel. Thus, P-hydrogel controls water movement and releases water in synchrony with crop needs. The enhanced water content in the soil profile in hydrogel applied plots might have improved soil physical conditions^17^. In concurrence with Fig. 6, P-hydrogel retained a higher amount of water compared to K-hydrogel and control (no-hydrogel) at both pressure points due to empty drainage pores and a larger proportion of soil capillary pores being filled with water, improving water availability to the crops in the long-run. Marginal but consistently higher soil moisture in P-hydrogel indicates a better soil–water regime, which could be of great significance when the soil moisture becomes limiting (limited and rainfed conditions), and therefore, facilitated higher root growth and yields.

The cost incurred and profitability was the two attributes that define the adoption of any new technology on a large scale. However, hydrogel applied plots (both P-hydrogel and K-hydrogel) recorded a higher cost of production (Table 3) due to the higher cost incurred on the hydrogels. Whereas, a significant increase in wheat profitability was found with the application of P-hydrogel due to higher grain yields compared to- hydrogel and K-hydrogel. Contrastingly, K-hydrogel at 5.0 kg ha^–1^ resulted in 3–16% lower net profitability over no-hydrogel. It could be associated with the higher cost of hydrogel but comparatively lower seed and grain yields in both test crops.

### Irrigation and hydrogel effects on productivity, root attributes and water productivity

Higher soybean yield in no-hydrogel plots with full and limited irrigations than hydrogel plots during 2017–2018 and 2018–2019, could be because the application of extra irrigations in the rainy season which coupled with sufficient rainfall could have caused excess water stress in the hydrogel applied plots, resulting in comparatively lower yields. While in rainfed plots, there was a significant increase in soybean yields in P-hydrogel and K-hydrogel over control (no-hydrogel). In wheat, full and limited irrigations coupled with P-hydrogel applied plots exhibited the highest yields over control and rainfed with P- and K-hydrogels. These results demonstrate that during sufficient rainfall years, the addition of P-hydrogel and K-hydrogel in absence of irrigation could produce superior soybean yields. In rabi season under less or negligible rainfall conditions, external application of irrigation enhances the water holding capacity of hydrogels, thus hydrogels release water as per the crop needs which resulted in higher yields in the respective plots. Equally, limited irrigations and rainfed plots with no-hydrogel registered higher wheat yield than K-hydrogel, could be due to lower soil moisture retention and higher release by K-hydrogel with an increase in soil matric suction resulted in loss of moisture through evaporation (Fig. 6).

### Implications of hydrogels application under zero tillage/conservation agriculture (CA)

Over the few decades due to excess tillage, input factor productivity of major crops and cropping systems have been declined. Thus, zero tillage (ZT) or no-tillage gained momentum in India and at the global level to reclaim depleted soil conditions and also to enhance input factor productivity. As a sustainable approach, the CA concept has been steadily increased globally with 124.8 M ha area^46^. In India, ZT/CA area expanded to 1.5 million hectares^47^ and expansion is less due to variable climatic conditions (rainfall dependent area is ~ 60%), soil type, and small landholdings. Hence, under CA/ZT situations, the application of hydrogels is presumed to be beneficial in enhancing crop productivity through enhanced soil moisture content. The major constraint under long-term residue retention ZT/CA fields is soil application of hydrogels and subsequent increase in the cost of hydrogel application. But hydrogel application methodologies like seed coating and slurry application enhance crop yield and water productivity significantly than soil application^48^. It is evident from the findings that crop yields enhanced at ~ 5.6% in Kharif and ~ 14.7% in with hydrogel application; it is presumed to produce additional ~ 7.34 million tons of food grains production (46% contribution from rainfed areas towards total grain production of 294 MT in India during 2020). Thus, hydrogel application is not only a viable sustainable crop production option under conventional tillage systems but it can be a good approach for yield enhancement under the ZT system in the Indo-Gangetic plains of India.

## Conclusions

The study proved the hypothesis that soil application of P-hydrogel led to significant improvement in soybean and wheat productivity, WP, rooting behavior and profitability of soybean–wheat cropping system in water stressed conditions. Thus, application of P-hydrogel resulted in significant improvement in soybean–wheat yields (20.2–22.4%), WP (6.8–41.1%), IWP (2.1–22.1%), and profitability (8.0–18.2%) over control plots (no hydrogel applied plots). Interestingly, P-hydrogel application with adequate irrigation exhibited higher yields. Thus adoption of P-hydrogel even in adequate or limited irrigated conditions further increases crop yields by reducing the number of irrigations or total water use. Therefore, the application of P-hydrogel with life-saving and precise irrigation management, a new management paradigm for scaling up the soybean-wheat system in north-west Indo-Gangetic Plains, can potentially help to address emerging challenges of water scarcity and sustainability in the water-scarce regions.

## Materials and methods

### Experimental site and weather conditions

The 2-year field experiment was conducted at the research farm of ICAR-Indian Agricultural Research Institute, New Delhi (28°40′ N 77°12′ E) in soybean–wheat system under conventional tillage system during 2017–2018 and 2018–2019. The experimental site has well-drained sandy loam alluvial soil type (Typic Haplustept) having sand (638 g kg^–1^), silt (167 g kg^–1^) and clay (195 g kg^–1^). The plow layer (0–15 cm) soil of the experimental field having 0.40% soil organic carbon, 137.5 kg ha^–1^ KMnO_4_ oxidizable N, 27.0 kg ha^–1^ NaHCO_3_ extractable phosphorus, 67.8 kg ha^–1^ 1.0 N NH_4_OAc exchangeable potassium and slightly alkaline (pH 8.4) (1:2.5 soil–water ratio)^49^. The initial experimental soils had 1.28 g cm^−3^ (0–15 cm) bulk density, 4.8 mm h^–1^ infiltration rate, 37.9, 8.6, and 29.3% (volume/volume, v/v) moisture content at field capacity, permanent wilting point, and available moisture content, respectively. The infiltration rates are the averaged infiltration rates over the period measured using double ring infiltrometer. Initial soil physical and chemical parameters are depicted in Table 4.

**Table 4:** Physical and chemical properties of the experimental soil.

Particulars	Values	Methods followed
**Mechanical analysis**		
Sand (%)	64.85	Hydrometer method^49^
Silt (%)	21.07	
Clay (%)	14.08	
Textural class	Sandy-loam	
**Physical properties**		
Bulk density (g cm^−3^)	1.28	Veihmeyer and Hendrickson method^49^
Infiltration rate (mm h^−1^)	4.8	Double ring infiltrometer method^49^
**Chemical properties**		
Soil organic carbon (%)	0.40	Walkley and Black method^49^
Available N (kg ha^−1^)	137.5	Alkaline permanganate method^49^
Available P (kg ha^−1^)	27.0	Olsen’s method^49^
Available K (kg ha^−1^)	67.8	Flame photometer method^49^
pH (1/2.5 soil:water ratio)	8.4	Beckman’s pH meter^49^

**Figure 8:**
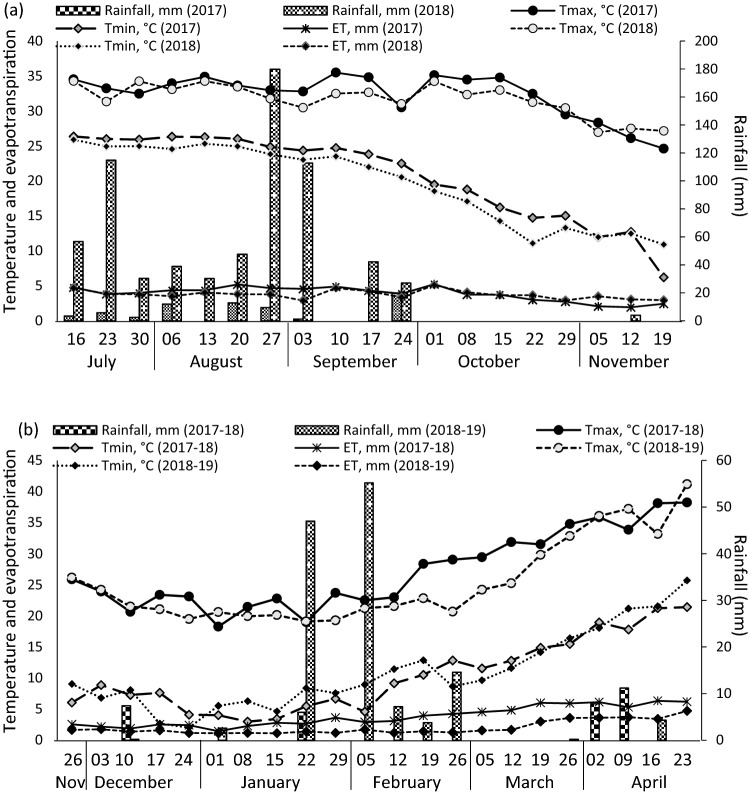
Weekly average temperature, evaporation (ET) and rainfall during (**a**) soybean and (**b**) wheat during 2017–2018 and 2018–2019 crop growing seasons.

The rainfall received during the soybean crop seasons (July 2017–October 2017, and July 2018–October 2018) was 803 and 919 mm, respectively. The first three months (July–September) witnessed > 80% of the total yearly rainfall during both years. The rainfall received during the winter season (October 2017–18 to April 2017–18, and October 2018–19 to April 2018–19) was 32.4 and 135.1 mm, respectively. The average weekly rainfall, weekly temperatures (T_max_ and T_min_), and weekly pan evaporation during study periods are depicted in Fig. 8a,b.

### Test materials and levels of application

P-hydrogel (Trade name: Pusa Jal Nidhi) was procured from M/S KCH India (P) Ltd, Chennai, India, and used as such. P-hydrogel is a cellulosic backbone cross linked with polyacrylate grafted. While, K-hydrogel was synthesized in the laboratory using derivative cellulose, kaolin, and acrylamide, by free radical polymerization technique^50^ with minor modifications. A mixture of bio-degradable cellulolytic derivatives and clay along with venyl monomers was added to warm water for the synthesis of K-hydrogel. A free radical initiator was added to the homogenized mixture with constant stirring. After a specific interval of time (6–12 h), the gel mass obtained was subjected to post reaction washing and drying to yield bio-polymeric grafted and cross-linked polyacrylate superabsorbent hydrogels. The salient characteristics of the two test hydrogels are given in Table 5. The two test hydrogels were applied as P-hydrogel @ 2.5 kg ha^–1^ and K-hydrogel @ 2.5 and 5.0 kg ha^–1^. These were applied manually after mixing with 2 mm sieved soil to make bulk. The entire dose of hydrogel were applied at the time of sowing at 5–6 cm soil depth in the seed zone in each crop season (i.e. 2 times each in soybean and wheat).

**Table 5:** Key characteristics of P- and K-hydrogels. Adopted from^50^.

Parameter	P-hydrogel	K-hydrogel
Chemical constitution	Derivatized cellulose based grafted and crosslinked anionic polyacrylate	Derivatized cellulose based grafted and crosslinked anionic polyacrylate incorporating kaolin
Appearance	Amorphous white/yellow granules	Amorphous brownish granules
Particle size	20–100 mesh (micro granules)	20–100 mesh (micro granules)
pH	7.0–7.5	7.0 – 7.5
Stability at 50 °C	Stable	Stable
Sensitivity to UV light	Not sensitive	Not sensitive
Maximum absorption in deionized water (50 °C)	350 g/g	600–800 g/g
Temperature of maximum absorption	50 °C	50 °C
Time taken for 60% swelling	2 h (approximately)	4 h (approximately)
Stability in soil	Less than 2 years (anticipated based on available literature)	Less than 2 years (anticipated based on available literature)
Toxicity in soil	None under normal conditions	None under normal conditions

### Experimental design and management practices

The field experiment was conducted in three replications using a split-plot design. The treatments included three irrigation regimes namely full irrigation (40% depletion of available soil moisture), limited irrigation (70% depletion of available soil moisture) where droughts stress created, and rainfed plots assigned in main plots. In rainfed plots, the necessary base irrigation (5.0 cm) was given in wheat to obtain a uniform crop stand as no rainfall received in the winter months for sowing of wheat. Sub-plots in each main plot comprised four treatments namely control (no hydrogel), K-hydrogel at 2.5 kg ha^–1^, K-hydrogel at 5.0 kg ha^–1^, and P-hydrogel at 2.5 kg ha^–1^ assigned randomly. The agronomic practices used in the study are listed in Table 6. All the sub-plots were of a uniform size of 16.2 m^2^ (4.5 × 3.6 m). To prevent peripheral water movement from irrigated plots to the rainfed plots, a 2-m wide buffer area was maintained.

**Table 6:** Agronomic practices followed and inputs applied during 2017–2019 in soybean–wheat system.

Agronomic practice	Soybean (2017 and 2018)	Wheat (2017–2018 and 2018–2019)
Variety/hybrid	PS 1347	HD 3086
Seed rate (kg ha^−1^)	80	100
Dates of sowing	15-07-2017; 09-07-2018	22-11-2017; 26-11-2018
Net plot size	3.6 × 2.5 m = 9.0 m^2^	3.6 × 2.5 m = 9.0 m^2^
Fertilizers (kg ha^−1^)	60:80:40:20 NPKS	100:60:40 NPK
Weed management	Pre-emergence: pendimethalin @ 0.75 kg a.i., 1 hand weeding at 40 DAS	Post-emergence: sulphosulfuron + metsulphuron @ 0.075 ml L^–1^ at 25 DAS
Dates of harvesting	01-11-2017; 26-10-2018	17-04-2019; 23-04-2019

*D* date, *M* month, *Y* year, *DAS* days after sowing.

The soybean genotype, PS 1347 with medium maturity duration (123 days) was sown during the first fortnight of July in both years at a spacing of 45 × 10 cm and harvested in the last week of October in each year. Wheat genotype, HD-3086 with long maturity duration (135 days) was sown on flatbeds at 22.5 × 5 cm row to row and plant to plant spacing in the last week of November and harvested in the last week of April in each year. Fertilizers @ 60:80:60 and 120:60:60 kg NPK ha^–1^ were applied in soybean and wheat, respectively. Urea [(NH_4_)_2_-CO], diammonium phosphate [(NH_4_)_2_HPO_4_], and potassium sulfate [K_2_SO_4_] were used as the sources of nutrients. The soybean field was kept weed-free by pre-emergence application of pendimethalin @ 0.75 kg ha^–1^ followed by one hand weeding (HW) at 40-days after sowing (DAS).

To reduce the weed problem, the post-emergence application of sulphosulfuron + metsulphuron @ 0.075 ml L^–1^ was done in wheat at 25 DAS. Based on the rainfall observed, 3 irrigations in 2017 and 2 irrigations in 2018 were given in full irrigation plots, and 2 irrigations in 2017 and 1 irrigation in 2018 in soybean were given under limited irrigation treatment. Similarly, in wheat under full irrigation plots 5 irrigation in 2017–18, and 3 irrigations in 2018–19 were given. While in limited irrigation plots, 4 irrigations in 2017–18 and 2 irrigations in 2018–19 were applied. In both the crops, each irrigation provided with a 5.0 cm equivalent of water using area × volume basis (Q = A × V), and scheduling was done at 40 and 70% depletion of available soil moisture (ASM = field capacity-permanent wilting point). Soil moisture content at field capacity and permanent wilting point was measured using pressure plate apparatus, where water retention at two specific soil water matric suction values (field capacity and wilting points) were observed. The conventional flood irrigation system was adopted to impose the irrigation treatments in both crops.

### Data collection and analyses

Roots were sampled at the anthesis stage by taking soil core at 0–30 cm soil depth, washed by placing them in nylon nets to remove soil debris and other extraneous materials. These were then scanned through an image scanner (Epson V700, Indonesia) and the length and volume were retrieved by using Win RHIZO version 5.0 (Regent Instruments Inc., Quebec City, Canada). The number of days when 50% of the wheat plant population reached a particular phenological stage was recorded as a period of attainment of that stage^51^. Plants were harvested from the net-plot area of 9.0 m^2^ (excluding 1.8 m and 0.3 m border area at both ends) in each plot to determine grain- and biomass-yields. After sun-drying for seven days, above-ground biomass from each plot was weighed, grains (~ 14% grain moisture) separated by a mechanical thresher, cleaned, and weighed.

### Soil water content measurements

Retention and release of water by the hydrogel amended soil samples were measured using a pressure plate apparatus (Soil moisture Equipment Corp., CA). Soils from the experimental site were mixed with hydrogels similarly as in the case of field applications. The soil was packed in small volumes (cylindrical; 5 cm diameter, 5 cm height) to the bulk densities as observed in the field, capillary-saturated, and subjected to 0.33 and 1 bar suction in pressure plate apparatus. For each suction, 5 replicates were used, and water retention was determined from the difference between weights of soil samples after equilibrium with each suction, and weights of the oven-dried soils thereafter. In pressure plate apparatus, a fully saturated soil sample is placed inside a sealed chamber, except for the bottom, which has a porous membrane exposed to atmospheric air pressure and upon which the sample is placed. A positive pressure of 15,000 hPa for Wilting Point and 330 hPa for Field Capacity is given to the chamber, until the membrane reaches equilibrium. After the sample has reached equilibrium, it is removed from the chamber and its mass is recorded. Finally, the dry mass is calculated, which is usually done by baking the soil for 24 h at 105 °C and then dry weight of the samples are measured. Then specific water content (kg water/kg soil) for field capacity and permanent wilting point can be calculated using Eqs. (1) and (2) as suggested by Richards^52^.

1 $${\text{WFC}} = \, \left( {{\text{M33}}0 - {\text{ Ms}}} \right) / {\text{Ms}}$$

2 $${\text{WPWP}} = \, \left( {{\text{M15}}000 - {\text{ Ms}}} \right)/{\text{Ms}}$$

where WFC is the specific water contents at field capacity (330 hPa), WPWP is the specific water content at permanent wilting point (15,000 hPa), M330 is the mass of the soil sample at 330 hPa, M15000 is the mass of the soil sample at 15,000 hPa, and Ms is the dry mass of soil.

The volumetric water content (volume/volume, v/v) was determined by converting gravimetric values with BD of soil (1.26 and 1.31 g cm^−3^, for 0–15 and 15–30 cm soil depths, respectively). For gravimetric soil moisture content estimation, soil samples were collected employing a soil auger (5.0 cm diameter) from 0–15 cm and 15–30 cm soil depths at the 10-day interval, water content determined on m/m basis of oven-dry soil. During 2018, volumetric water content (v/v) was determined using a sensor-based soil moisture meter (Diviner-2000) with moisture access probes of 1.0 m length at 10-day intervals, and values are depicted for 0–10, 10–20, 20–30, and 30–40 cm soil profile depths.

### Water productivity (WP) and irrigation water productivity (IWP) estimations

WP of the crops under different treatments was computed by dividing the grain yield (kg ha^–1^) with the amount of irrigation water (cm) and effective rainfall (Rainfall more than 6.25 mm on any day is considered as ineffective and it should be multiplied with 0.65 to get effective rainfall, https://www.fao.org/3/x5560e/x5560e03.htm) from the respective plots as per the Eq. (3)^53^:

3 $${\text{WP }}\left( {{\text{kg ha cm}}^{{ - {1}}} } \right) = {\text{ Ye / }}\left\{ {{\text{Iw }} + {\text{ Er}}} \right\}$$

where, WP is the water productivity (kg ha cm^−1^), Ye is the economic yield (seed/grain, kg ha^−1^), Iw is the irrigation water applied (cm), Er is the effective rainfall (cm).

Irrigation water productivity (IWP) was computed by dividing the grain yield (kg ha^–1^) by irrigation water applied (cm) from the respective plots^53^ (Eq. 4):

4 $${\text{IWP }}\left( {{\text{kg ha cm}}^{{ - {1}}} } \right) = {\text{ Ye }}/{\text{ Iw}}$$

where, IWP is the irrigation water productivity (kg ha cm^−1^), Ye is the economic yield (seed/grain, kg ha^−1^), Iw is the irrigation water applied (cm).

### Yield measurements

The productive capacity of the soybean-wheat systems under different irrigation regimes and hydrogels was measured in terms of wheat grain equivalent yield (WGEY) at a price scale. The WGEY was calculated by using the Eq. (5)^53^

5 $${\text{WGEY }}\left( {{\text{Mg ha}}^{{ - {1}}} } \right) = {\text{ Yw }} + \, \left\{ {\left( {{\text{Ys }} \times {\text{ Ps}}} \right)/{\text{ Pw}}} \right\}$$

where, WGEY is the wheat grain equivalent yield (Mg ha^−1^), Yw is the wheat grain yield (Mg ha^−1^), Ys is the soybean seed yield (Mg ha^−1^), Pw is the price of wheat grain (US$ Mg^−1^) and Pw is the price of wheat grain (US$Mg^−1^).

### Farm profitability analysis

The relative economics of treatments was calculated using the minimum support price of soybean and wheat declared by the Government of India during 2017–2018 and 2018–2019, and costs incurred in operations involved in raising crops from field preparation to harvesting and storage. Since no cost is incurred on the amount of irrigation water in India, only cost incurred on irrigation water application at a rate of US$ 5.33 per irrigation has been used to calculate the cost of irrigation. The cost of K-hydrogel and P-hydrogel was US$11.97 kg^–1^ and US$12.68 kg^–1^, respectively. Profitability (US$ ha^–1^) was calculated by adding the yearly net profitability of soybean and wheat during both the study years. The minimum support price (MSP) for soybean was US$ 429.58 Mg^–1^ in 2017–18 and US$ 478.73 Mg^–1^ in 2018–2019. The MSP for wheat was US$ 244.37 and US$ 259.15 Mg^–1^, respectively.

### Statistical analyses

Soybean and wheat growth and yield data recorded during the two years were analyzed through the analysis of variance (ANOVA) test for a split-plot design as suggested by Gomez and Gomez^54^ using SAS 9.3 software (SAS Institute, Cary, NC). The significance of the difference between the main plots (soil moisture levels), sub-plots (hydrogels), and interaction effect of soil moisture levels and hydrogels was tested. Means were compared by Duncan’s Multiple Range Test (DMRT).

Authors have confirmed that, all plant investigations were carried out in accordance with appropriate national, international, or institutional guidelines.
